# Thrombospondin-1 Silencing Down-Regulates Integrin Expression Levels in Human Anaplastic Thyroid Cancer Cells with BRAF^V600E^: New Insights in the Host Tissue Adaptation and Homeostasis of Tumor Microenvironment

**DOI:** 10.3389/fendo.2013.00189

**Published:** 2013-12-02

**Authors:** Mark Duquette, Peter M. Sadow, Jack Lawler, Carmelo Nucera

**Affiliations:** ^1^Human Thyroid Cancers Preclinical and Translational Research Laboratory, Division of Cancer Biology and Angiogenesis, Department of Pathology, Harvard Medical School, Beth Israel Deaconess Medical Center (BIDMC), Boston, MA, USA; ^2^Endocrine Service, Department of Pathology, Massachusetts General Hospital, Harvard Medical School, Boston, MA, USA; ^3^Division of Cancer Biology and Angiogenesis, Department of Pathology, Harvard Medical School, Center for Vascular Biology Research (CVBR), Beth Israel Deaconess Medical Center (BIDMC), Boston, MA, USA

**Keywords:** BRAF^V600E^, integrins, thyroid cancer, microenvironment, extracellular matrix, TSP-1

## Abstract

**Background and Rationale**: Anaplastic thyroid cancer (ATC) is characterized by pleomorphic cells, has a poor prognosis, is highly devastating disease, and is not curable. No reliable biomarkers of metastatic potential, helpful for early diagnosis of ATC and therapeutic response have been found yet. Thrombospondin-1 (TSP-1) plays a fundamental role in cancer progression by regulating cell stromal cross-talk in the tumor microenvironment.

**Goals**: Our goal was to understand whether TSP-1 could affect protein levels of its integrin receptors (e.g., ITGα3, α6, and β1) and cell morphology in BRAF^V600E^-ATC cells *in vitro* and *in vivo*.

**Experimental Design**: Anaplastic thyroid cancer-derived cell cultures and western blotting were used to assess integrin protein expression upon TSP-1 silencing. Immunohistochemistry was performed on orthotopic primary human ATC and metastatic ATC in lung tissue to compare TSP-1 and integrin protein expression levels.

**Results**: TSP-1 knock-down down-regulates ITGα3, α6, and β1 in BRAF^V600E^-human ATC cells. BRAF^V600E^-ATC cells with TSP-1 knock-down were rounded compared to control cells, which displayed a spread morphology. TSP-1 knock-down also reduced TSP-1, ITGα3, α6, and β1 protein expression levels *in vivo* in the ATC microenvironment, which is enriched in stromal and inflammatory cells.

**Conclusion**: TSP-1 silencing causes changes in ITG levels and ATC cell morphology. The assessment of TSP-1 and ITG levels might contribute to earlier metastatic potential of BRAF^V600E^-positive aggressive thyroid cancers, and allow improved patient selection for clinical trials.

## Introduction

The incidence of thyroid cancer is increasing more rapidly than other cancers in the US (1) and in other countries (2). Anaplastic thyroid cancer (ATC) has perhaps the worst prognosis of any cancer, with a median survival of about 5 months and a 20% 1-year survival rate (3). ATC is resistant to standard chemotherapy, external beam radiation, and radioiodine treatment (3), thus new treatments are urgently needed. Outcomes could be improved with routine assessment of pro-metastatic biomarkers, which could enable earlier metastatic potential of this type of fatal thyroid cancer. The BRAF^V600E^ mutation is the most prevalent genetic alteration (greater than 50%) in papillary thyroid cancer (PTC) and is implicated in the progression of PTC to ATC (4–6), a crucial challenge in thyroid cancer. Our previous studies demonstrated the pro-metastatic role of the secreted extracellular matrix (ECM) protein thrombospondin-1 (TSP-1) in BRAF^V600E^-positive PTC (5, 7, 8) and indicated that TSP-1 increased phosphorylation of ERK1/2 (5). Gene Set Enrichment Analysis (GSEA) (5) was performed on a cohort of BRAF^V600E^ or BRAF^WT^ PTC specimens and normal thyroid tissue (NT) samples. We found 18 independent gene sets (of 539 tested) significantly associated with BRAF^V600E^ PTCs: 17 up-regulated and 1 down-regulated set (5). The GSEA data revealed that TSP-1 and several integrins were up-regulated in the BRAF^V600E^-positive human PTC (5).

TSP-1 binds to a wide variety of integrins, however the best characterized are integrin alpha3/beta1 and alpha6/beta1 (ITGα3/ITGβ1 or ITGα6/ITGβ1) (9–11). TSP-1 also binds non-integrin cell surface receptors (i.e., proteoglycans, CD36, CD47), matrix proteins [i.e., Fibronectin (FN)], cytokines (i.e., TGF-β1), pro-angiogenic factors (e.g., VEGF), and matrix proteases (i.e., MMP-9), indicating its importance in cross-talk between ECM molecules and their receptors (11, 12). Also, TSP-1 is involved in tumor cell adhesion and migration, and it may direct clustering of receptors to specialized domains for these biological processes (10). Integrins are a family of cell surface glycoproteins that function as receptors for ECM proteins, mediating both cell–cell and cell-ECM adhesion. Integrins are non-covalent, heterodimeric complexes of an alpha (α) and a beta (β) subunit (13). Their role is fundamental in cell microenvironment homeostasis, including either physiological or pathological conditions. Whereas, the role of TSP-1 in angiogenesis is well documented, its role in tumor metastasis is only just emerging. TSP-1 has been shown to promote metastasis in a breast cancer model (14). Our previous study has shown that the N-terminal domain of TSP-1 is involved in BRAF^V600E^-mediated invasion in thyroid cancer cells (5). Chandrasekaran et al. (10) also showed a critical role for the TSP-1 N-terminal domain in breast cancer cell invasion via putative binding site(s) to ITGα3/ITGβ1, which has an important role in tumor cell migration and invasion. Sumimoto et al. (15) have shown that BRAF^V600E^ knock-down decreased phospho-ERK1/2 protein levels and inhibited invasion of melanoma cells accompanied by a decrease of matrix metalloproteinase activity and ITGβ1 expression. Dynamic and reciprocal interactions involving cell adhesion molecules (e.g., integrins, CD44), ECM non-cellular components (i.e., TSP-1, FN), and soluble cytokines occur between tumor epithelial cells and tumor microenvironment stromal cells (13). Importantly, TSP-1 could be a valid biomarker for PTC aggressiveness and we have already established an immunohistochemistry (IHC)-based screening assay suitable for clinical trials (5). The goal of this brief research article is to understand whether TSP-1 affects integrin levels and cell morphology in BRAF^V600E^-positive ATC cells, contributing to metastasis.

## Materials and Methods

### Cell culture

The SW1736 ATC cell line, which harbors heterozygous BRAF^WT/V600E^, was kindly provided by N. E. Heldin (University of Uppsala, Uppsala, Sweden). The 8505c ATC cell line homozygous for BRAF^V600E/V600E^ was purchased from DSMZ (German collection of microorganisms and cell culture) (Brunswick, Germany). The 8505c cell line was established by Dr. M. Akiyama (Radiation Effects Research Foundation, Hiroshima, Japan) from the primary tumor of a 78-year-old woman with undifferentiated carcinoma. It is histologically an ATC with some spindle, polygonal, and giant cells (data by DSMZ). SW1736 and 8505c cell lines were grown in RPMI 1640 medium supplemented with 10% fetal bovine serum and penicillin/streptomycin/amphotericin.

### Antibodies

Antibodies against the following proteins were used: β-actin (A-5316) (Sigma); TSP-1 (A6.1) for IHC (Abcam, USA), and the previously validated TSP-1 clone MA-I for Western blot (5, 16) and R1 for immunofluorescence (17, 18); anti-ITGα3 (C-18, Santa Cruz Biotechnology, USA), anti-ITGα6 (H-87, Santa Cruz Biotechnology, USA), and anti-ITGβ1 (kindly provided from Dr. Richard Hynes, MIT, Cambridge, MA, USA); anti phospho-FAK (cat. #3283, Cell Signaling, USA), and anti-total FAK (cat #3285, Cell Signaling, USA). CD45 (cat#550539, BD Pharmingen, USA), F4-80 (cat# 14-4801, eBioscience, USA), and αSMA (alpha-smooth muscle actin) (A2547, Sigma, USA).

### Cell transfections for lentivirus production

HEK 293T cells (5 × 10^5^) were grown in 60-mm plates and transfected using Fugene-6 (Roche) in OptiMEM (Invitrogen) for 48 h according to the manufacturer’s instructions.

### TSP-1 silencing techniques

Stable transduced [shRNA (sh) viral transductions] ATC cells with or without TSP-1 knock-down were established according to Nucera et al. (5).

### Western blot

Western blotting assays were performed following standard protocols; cells were lysed in buffer, composed of 10 mM Hepes (pH 7.40), 150 mM NaCl, 5 mM EDTA, 1 mM EGTA, 1 mM sodium vanadate, 5 mM sodium fluoride, and 1% Triton-X 100; protease and phosphatase inhibitors (Pierce) were used for protein extractions (5).

### *In vivo* studies

The animal work was done in the animal facility at Beth Israel Deaconess Medical Center (Boston, MA, USA) in accordance with federal, local, and institutional guidelines. We used an orthotopic mouse model of ATC as previously described and validated by Nucera et al. (19) (5), using female about 6-week-old severe combined immunodeficient (SCID) mice (Taconic, USA).

### Histology and immunohistochemistry

All tissue specimens (five primary orthotopic ATC or lung specimens from mice with sh-control TSP-1 8505c cells; and five primary orthotopic ATC or lung specimens from mice with sh-TSP-1 knock-down 8505 cells) were fixed with 10% buffered formalin phosphate and embedded in paraffin blocks. Histopathology evaluation was performed by a pathologist (Peter M. Sadow) on hematoxylin and eosin-stained tissue sections of orthotopic 8505c ATC specimens (5). All photos were captured with an Olympus BX41 microscope and the Olympus Q COLOR 5 photo camera (Olympus Corp., Lake Success, NY, USA), using the Twain software in Adobe Photoshop (7.0) and white balanced with the same method for all images. Sections (4 μm thick) of formalin-fixed orthotopic 8505c ATC specimens (5) were used for IHC procedures. After baking overnight at 37°C, deparaffinization with xylene/ethanol and rehydration were performed. IHC analysis was performed using primary antibodies against human TSP-1 (1:25, citrate buffer for antigen retrieval), ITGα3 (1:250, citrate buffer for antigen retrieval), ITGα6 (1:200, citrate buffer for antigen retrieval), or ITGβ1 (1:500, citrate buffer for antigen retrieval); anti-mouse CD45 antibody (#550539, BD Pharmingen, USA): 1:50, citrate buffer and pressure cooker for antigen retrieval; anti-mouse F4-80 antibody (pan macrophage marker) (#144801, eBioscience, USA): 1:50, citrate buffer and pressure cooker for antigen retrieval; and anti-mouse alpha-smooth muscle actin (αSMA) (#A-2547, Sigma, USA) (1:20,000). The sections, treated with pressure cooker for antigen retrieval (Biocare Medical, Concord, CA, USA), were incubated at 123°C in citrate buffer (Dako Target Retrieval Solution, S1699; DAKO Corp.), cooled and washed with PBS. Antigen retrieval was performed for 60 min at room temperature. The primary antibody was detected using a biotin-free secondary antibody (K4011) (Dako Envision system) and incubated for 30 min. All incubations were carried out in a humid chamber at room temperature. Slides were rinsed with PBS between incubations. Sections were developed using 3,3-diaminobenzidine (Sigma Chemical Co.) as a substrate and were counterstained with Mayer’s hematoxylin. PAX8 and p53 immunostaining was performed according to our previous study (19). We used specie-specific IgG as negative control. The IHC markers were assessed semiquantitatively using the following scoring method: 0 negative, 1 1–10% positive cells (low expression), 2 11–50% positive cells (moderate), and 3 more than 50% positive cells (high expression) according to Shaik et al. (20).

### Immunofluorescence

For immunofluorescence experiments, 5 × 10^4^ 8505c or SW1736 cells were seeded on type I collagen-coated cover slips (BD Biosciences) for 24 h. Cells were washed three times with PBS, fixed with 4% paraformaldehyde for 10 min at room temperature, and permeabilized with PBS 0.5% Triton-X 100 for 5 min at room temperature. After three washes with PBS, cells were blocked with TBST 1% BSA for 20 min, followed by incubation with phalloidin-fluorescein (Sigma, USA) in PBST 1% BSA for 30 min at room temperature. Cells were rinsed three times with TBS. Finally, the cover slips were mounted with a mixture of Vectashield mounting medium and DAPI (Vector Laboratories). Cells were imaged at 20× on a Nikon Eclipse 300 epifluorescence inverted microscope connected to a Retiga 2000RV camera (Nikon Instruments).

### Statistical analysis

Statistical analysis was carried out using Microsoft Excel Software. Results were compared using the Student’s *t*-test and χ^2^ test. *P* values of <0.05 were considered significant (*P < 0.05, **P < 0.01, ***P < 0.001). Densitometry analysis was performed using Quantity One software (BioRad, USA).

## Results

### Knock-down of TSP-1 down-regulates integrins levels and affects cell morphology in human anaplastic thyroid cancer cells with BRAF^V600E^

**Figure 1:**
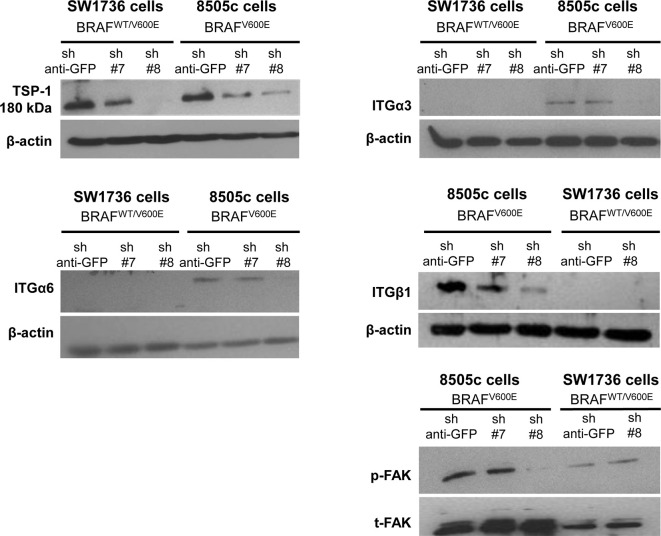
**TSP-1, ITGα6, ITGα3, ITGβ1, and pFAK protein levels by western blot in human anaplastic thyroid cancer (ATC) cell lines with sh-GFP (green fluorescent protein, control) or knock-down of TSP-1 (#7 and #8) harboring heterozygous BRAF^WT/V600E^ (SW1736 cells) or homozygous BRAF^V600E^ (8505c cells)**.

**Figure 2:**
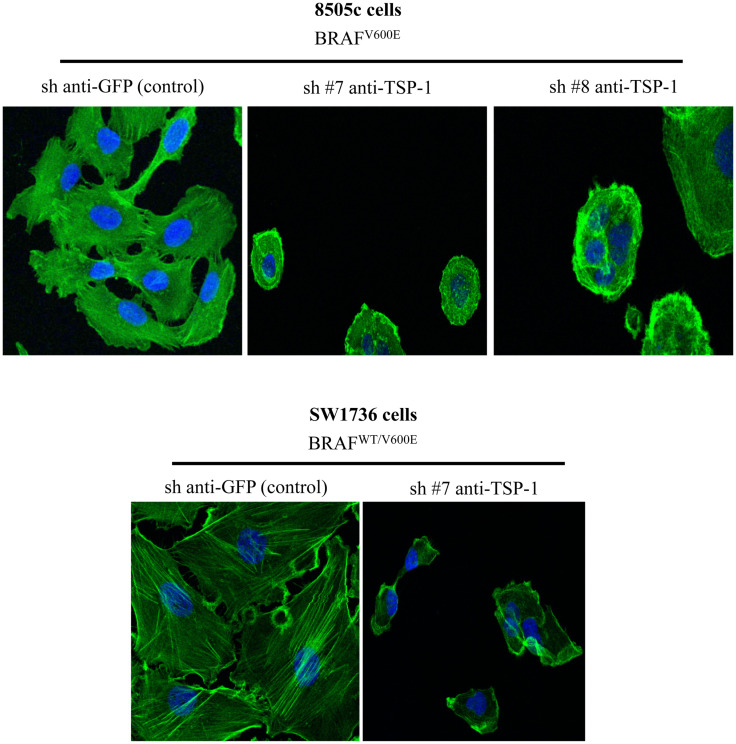
**Phalloidin staining in the control (sh-GFP) 8505c human anaplastic thyroid cancer (ATC) cells or in the 8505c ATC cells with TSP-1 knock-down (sh-TSP-1), and in the sh-GFP (control) SW1736 ATC cells or in the TSP-1 knock-down (sh-TSP-1) SW1736 ATC cells**. The cells were plated on type I collagen-coated cover slips (Magnifications: 20×). Sh, short hairpin RNA used for knock-down.

Our results show that ITGα3 (∼95%), ITGα6 (∼95%), and ITGβ1 (∼90%) subunits are decreased in homozygous BRAF^V600E^-positive 8505c ATC cells with TSP-1 knock-down (Figure 1). Furthermore, either homozygous BRAF^V600E^-positve 8505c ATC cells or heterozygous BRAF^V600E^-positive SW1736 ATC cells with TSP-1 knock-down display a markedly different morphology (rounded cells) compared to sh-controls cells (spread morphology) when plated on type 1 collagen (Figure 2). We also found that TSP-1 knock-down by shRNA (sh) caused a down-regulation of pFAK protein levels (∼90%) in human thyroid cancer cells with homozygous BRAF^V600E^ (Figure 1).

### TSP-1 knock-down down-regulates integrins levels in the orthotopic BRAF^V600E^ metastatic 8505 ATC cells *in vivo*

**Figure 3:**
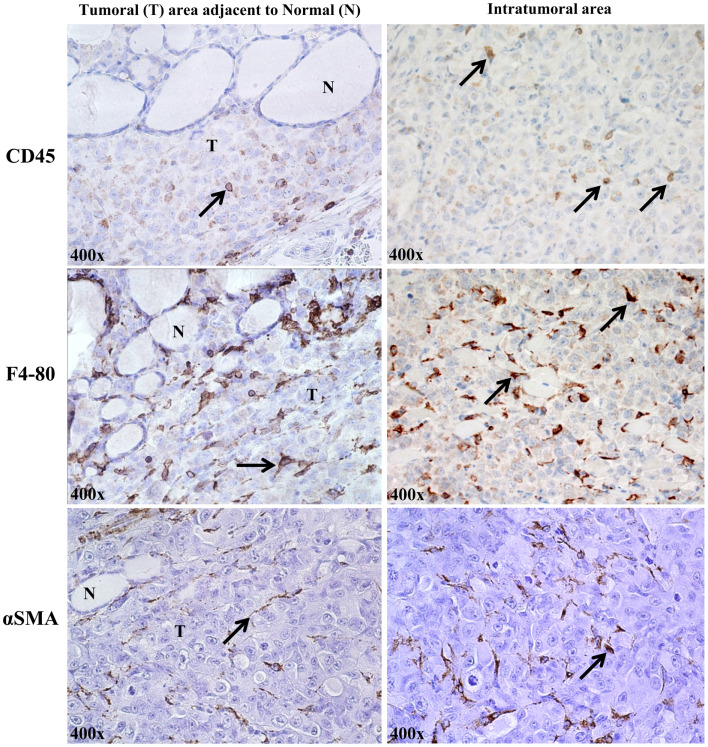
**Stromal cells in the orthotopic 8505c human anaplastic thyroid cancer (ATC) microenvironment of SCID immunocompromised mice (400× magnification, representative of eight mice)**. About 2% of cells/field were of lymphocytic lineage (CD45, localized to plasma membrane); ∼8% were identified as macrophages [F4/80 (pan macrophage marker), localized to plasma membrane]; and ∼5% were spindle-shaped pericytes, identified by staining for cytosolic αSMA (alpha-smooth muscle actin).

The orthotopic human BRAF^V600E^-positive 8505c ATC microenvironment *in vivo* shows stromal cells, identified by expression of some marker proteins: CD45+ (lymphocytic lineage), F4/80+ (macrophages) (21, 22), and αSMA+ (spindle-shaped pericytes) (23) (Figure 3).

**Figure 4:**
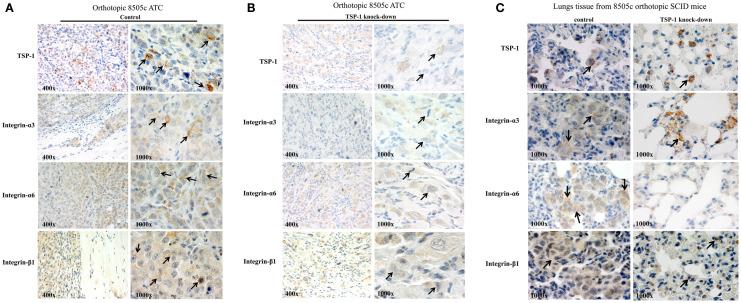
**TSP-1, ITGα3, ITGα6, and ITGβ1 immunoexpression in the orthotopic 8505c human anaplastic thyroid cancer (ATC) (A) without TSP-1 knock-down (control, arrows highlight protein immunoexpression in the 8505c ATC cells *in vivo*) or with TSP-1 knock-down (arrows highlight protein immunoexpression in the 8505c ATC cells *in vivo*) and (B) with TSP-1 knock-down (control, arrows highlight protein immunoexpression in the 8505c ATC cells *in vivo*)**. **(C)** Orthotopic 8505c metastatic ATC cells in the lungs of SCID mice; arrows mark protein immunoexpression of TSP-1, ITGα3, ITGα6, and ITGβ1 in the metastatic 8505c cells in the lungs tissue of control SCID mice. By contrast, IHC staining in the TSP-1 knock-down condition highlights TSP-1, ITGα3 and ITGβ1 protein expression in macrophages (noted by the dot-like, granular cytoplasmic staining, and bland histomorphology) but not in the metastatic 8505c cells which were absent; whereas, ITGα6 protein immunoexpression is completely negative in the TSP-1 knock-down condition.

The 8505c cells with TSP-1 knock-down metastasize less (5) and show very low protein levels of (i) ITGα3 [from 10 ITGα3-positive cells (score 2) in the sh-control 8505 orthotopic tumors to 2 cells ± 0.5/field in the sh-TSP-1 8505c orthotopic tumors, score: 1+, *P* < 0.05]; (ii) ITGα6 [from 16 ITGα6-positive cells (score 2) in the sh-control 8505 orthotopic tumors to 3 cells ± 0.35/field; score: 1+, *P* < 0.05]; and (iii) ITGβ1 [from 12 ITGβ1-positive cells (score 2) in the sh-control 8505 orthotopic tumors to 2 cells ± 0.42/field; score: 1, *P* < 0.05] in the primary orthotopic 8505c ATC (Figures 4A,B), as well as in the lungs (Figure 4C) where generally 8505c ATC cells preferentially metastasize (19). IHC staining in the TSP-1 knock-down condition (Figure 4C) highlights TSP-1 and integrin protein expression in macrophages (noted by the dot-like, granular cytoplasmic staining, and bland histomorphology) but not in the metastatic 8505c cells. Additionally, lung tissue from the 8505c ATC orthotopic mice with TSP-1 knock-down was also completely negative for both PAX8 and p53 protein expression that show prominent nuclear staining in 8505c cells which were absent (data not shown) (19). Furthermore, we have also found that TSP-1 [3 cells positive/field (score 2)], ITGα3 [6 cells positive/field (score 2)], and ITGβ1 [2 cells positive/field (score 1)] proteins were expressed in the metastatic 8505c cells in the lungs but not up-regulated compared to the their expression in the primary orthotopic 8505c human ATC cells (Figure 4), thus suggesting that the potential intravasation and colonization of ATC cells do not up-regulate the basal protein expression levels of TSP-1, ITGα3, and ITGβ1, which might be sufficient to trigger metastasis. By contrast, we found that ITGα6 protein expression levels [2 cells positive/field (score 2)] were up-regulated in the orthotopic metastatic 8505c ATC cells in the lungs compared to the primary orthotopic 8505c ATC cells in the mouse thyroid.

## Discussion

Thyroid carcinomas are increasing in frequency and account for 2.5% of all cancers in the United States (USA). ATC is a devastating disease with low survival rate due to uncontrolled systemic metastasis and with no effective treatment options. ATC represents 1.7% of all human thyroid cancers in USA, and geographically its prevalence ranges from 1.3 to 9.8% (median = 3.6%) (3). Conventional ATC therapy currently utilizes a multimodal approach using radiation therapy and chemotherapeutic agents such as doxorubicin. The local disease burden often is so extensive that surgery is confined to tumor debulking and securing the airway. Additionally, no reliable biomarkers of metastatic potential, helpful for early diagnosis of ATC and therapeutic response have been found yet.

ATC shows a pleomorphic and interconnected network between high-grade malignant cells and stromal cells (e.g., macrophages) that characterize tumor microenvironment (24). Here our results from an *in vivo* orthotopic mouse model of human ATC also suggest that the ATC microenvironment is enriched in stromal cells, including macrophages, leukocytes, endothelial cells, and pericytes, which might contribute to the ATC aggressiveness.

Changes in the tumor microenvironment (e.g., deregulation of ECM molecules or ECM receptors) are a critical step in human cancer invasion, metastasis, and progression. We found that TSP-1 (a key player for ECM remodeling) and integrins (ECM receptors) are associated with BRAF^V600E^-positive PTC (5). We also showed that BRAF^V600E^ or TSP-1 knock-down (5) down-regulated ERK1/2 phosphorylation protein levels and inhibited ATC cell proliferation adhesion, migration, and invasion, all properties associated with integrin-mediated interactions with the ECM.

TSP-1 is a secreted/soluble protein that can be assessed in the plasma of patients with breast cancer, and shows significantly higher plasma concentrations than normal individuals or patients with benign breast disease (25, 26). It plays an important role in the physiology and pathology of the cell microenvironment (7, 12, 27, 28), and it has been proposed to have both pro-metastatic and anti-metastatic properties (14, 29). TSP-1 in the mammary tumor microenvironment inhibits angiogenesis and breast cancer growth, but promotes metastasis to the lung in a transgenic model of breast cancer (14). The ability of TSP-1 to support metastasis correlates with its ability to promote tumor cell migration (14).

Integrins are TSP-1 receptors that mediate tumor cell-ECM adhesion and provide both the connection to the adhesive substrate and cellular signaling crucial for cell proliferation, migration, and invasion (9, 13). To our best knowledge, this is the first report that shows that TSP-1 affects protein levels of integrins in BRAF^V600E^-positive human ATC cells *in vitro* and *in vivo*. Also, TSP-1 knock-down *per se* significantly alters ATC cell morphology on type 1 collagen. Collectively, our results may suggest that BRAF^V600E^-ATC cells might acquire an adaptation in the host tissue during their tissue colonization, reprogram their gene expression profile and up-regulate integrin (i.e., ITGα6) protein levels. This observation is supported from a recent report that shows that TSP-1 stimulates ITGα6 protein expression levels in human breast carcinoma cells, promoting tumor cell adhesion and invasion (9). Additionally, the missense SNP rs11895564 (Ala380Thr) in ITGα6 may be a risk factor of thyroid cancer, contributing to the progression of PTC (30). Also, our results suggest that the zygosity (e.g., homozygous vs. heterozygous allelic mutations) of the BRAF^V600E^ mutation represents an important factor to take under consideration as a molecular modulator of the expression levels of integrins. In fact, the expression levels of many target genes could depend on the oncogenic dosage in human cancer cell. However, further studies are needed to understand better this aspect and the molecular role of integrins in thyroid cancer metastasis and progression.

Furthermore, integrins can also activate the FAK signaling cascade and promote PI3K kinase activity, which is essential to promote cancer invasion (31). Here, our results may suggest that TSP-1 protein levels also affect phospho-FAK protein levels, highlighting that TSP-1 might not only stimulate the ERK1/2 phosphorylation but additionally drive thyroid cancer cell adhesion and migration through FAK pathway(s). Shibue and Weinberg (31) demonstrated that ITGβ1 is fundamental to activate FAK signaling axis in controlling the initial proliferation of micro-metastatic mouse breast cancer cells disseminated in the lungs (31).

Overall, these results suggest that TSP-1 is a pro-metastatic constituent and TSP-1 silencing causes changes in integrin expression levels and ATC cell morphology. Therapeutic strategies aimed at modulating the thyroid cancer microenvironment might provide an additional perspective for the treatment of patients with these types of cancers. Routine assessment of pro-metastatic biomarkers, including TSP-1 and integrins, will help monitor patients undergoing targeted therapies, enable earlier metastatic potential of aggressive BRAF^V600E^-positive human thyroid cancer, foster development of innovative therapies for refractory thyroid cancer to current treatments, and allow improved patient selection for clinical trials.

## Conflict of Interest Statement

The authors declare that the research was conducted in the absence of any commercial or financial relationships that could be construed as a potential conflict of interest.
