# Corrigendum: Thrombospondin-1 Silencing Down-Regulates Integrin Expression Levels in Human Anaplastic Thyroid Cancer Cells With BRAF^V600E^: New Insights in the Host Tissue Adaptation and Homeostasis of Tumor Microenvironment

**DOI:** 10.3389/fendo.2020.596223

**Published:** 2020-10-09

**Authors:** Mark Duquette, Peter M. Sadow, Jack Lawler, Carmelo Nucera

**Affiliations:** ^1^Human Thyroid Cancers Preclinical and Translational Research Laboratory, Division of Cancer Biology and Angiogenesis, Department of Pathology, Harvard Medical School, Beth Israel Deaconess Medical Center (BIDMC), Boston, MA, United States; ^2^Endocrine Service, Department of Pathology, Massachusetts General Hospital, Harvard Medical School, Boston, MA, United States; ^3^Division of Cancer Biology and Angiogenesis, Department of Pathology, Harvard Medical School, Center for Vascular Biology Research (CVBR), Beth Israel Deaconess Medical Center (BIDMC), Boston, MA, United States

**Keywords:** BRAF^V600E^, integrins, thyroid cancer, microenvironment, extracellular matrix, TSP-1

In the original article, there was a mistake in Figure 1 as published. The authors note a mislabeling of the cell lines name which appeared in Fig.1 left upper panel and have corrected it, andthe loading control was replaced. This correction is not of scientific nature and does not change results and conclusions of the article. The corrected Figure 1 appears below.

The authors apologize and state that this does not change the scientific conclusions of the article in any way. The original article has been updated.

**Figure 1:**
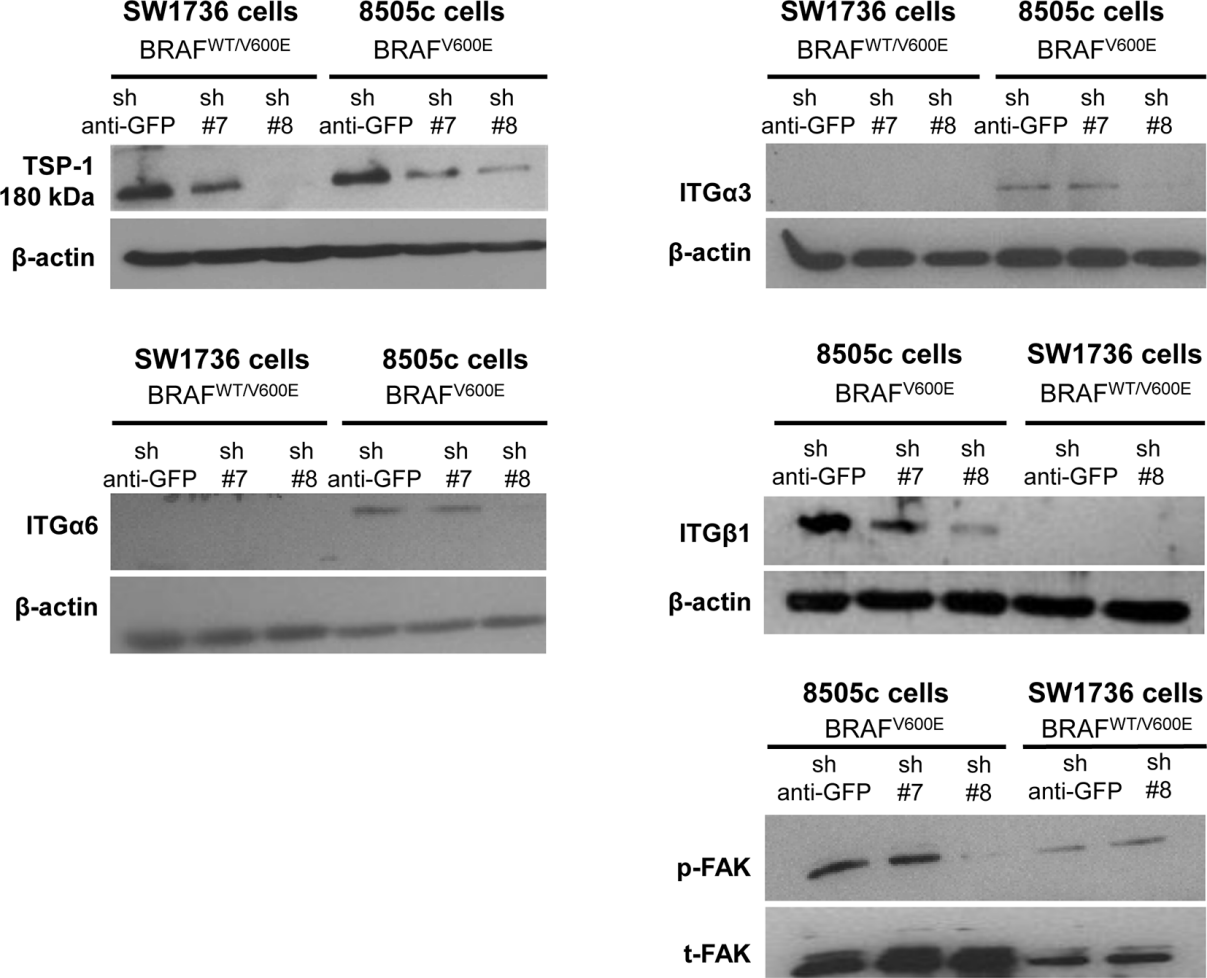
TSP-1, ITGα6, ITGα3, ITGβ1, and pFAK protein levels by western blot in human anaplastic thyroid cancer (ATC) cell lines with sh-GFP (green fluorescent protein, control) or knock-down of TSP-1 (#7 and #8) harboring heterozygous BRAF^WT/V600E^ (SW1736 cells) or homozygous BRAF^V600E^ (8505c cells).

